# 4578 Early life stress promotes chronicity of experimental colitis

**DOI:** 10.1017/cts.2020.65

**Published:** 2020-07-29

**Authors:** Rachel Quinn Muir, Barbara J. Klocke, Kasi C. McPherson, Jeremy B. Foote, Jennifer S. Pollock, Craig L. Maynard

**Affiliations:** 1 University of Alabama at Birmingham

## Abstract

OBJECTIVES/GOALS: The overall goal of this study was to determine the effect of early life stress (ELS) on the intestinal CD4+ T cell immune compartment, at homeostasis and after induction of experimental Inflammatory Bowel Disease (IBD). METHODS/STUDY POPULATION: We used a mouse model of ELS, maternal separation with early weaning (MSEW). We used IL-10 reporter mice to enable analysis of IL-10-producing cells. Mice were examined on postnatal day 28 to determine the impact of ELS on gut regulatory T cells. Plasma levels of corticosterone (rodent stress response hormone) was determined by ELISA. Colitis was induced in MSEW and normal rear (NR) mice via intraperitoneal injection of α-IL-10R every 5 days until day 15. Mice were euthanized on days 20 and 30. Colonic tissue sections were stained for histological analysis. Remaining tissue was further processed for flow cytometric analysis of CD4+ T cells and innate lymphoid cells. RESULTS/ANTICIPATED RESULTS: Plasma corticosterone was elevated in MSEW mice compared to their NR counterparts at 4 weeks of age. We observed that the MSEW stress protocol does not affect the baseline colonic CD4+ T cell or innate lymphoid cell populations. There was a reduction in the intestinal CD4+ T cells and regulatory T cells on day 20 in α-IL-10R MSEW mice compared to NR counterparts. This difference disappeared by day 30. Histological scoring showed no difference in disease severity between α-IL-10R treated MSEW and NR mice on day 20. However, on day 30, when α-IL-10R NR mice are recovering from colitis, MSEW mice showed persistent histological inflammation, mainly attributable to sustained epithelial damage. DISCUSSION/SIGNIFICANCE OF IMPACT: Our results suggest that ELS prolongs intestinal inflammation and impairs epithelial repair. Future studies will focus on elucidating the mechanisms responsible for ELS-dependent impairment of mucosal repair in experimental colitis.

